# Associations of intracranial artery length and branch number on non-contrast enhanced MRA with cognitive impairment in individuals with carotid atherosclerosis

**DOI:** 10.1038/s41598-022-11418-y

**Published:** 2022-05-06

**Authors:** Zhensen Chen, Anders Gould, Duygu Baylam Geleri, Niranjan Balu, Li Chen, Baocheng Chu, Kristi Pimentel, Gador Canton, Thomas S. Hatsukami, Chun Yuan

**Affiliations:** 1 Vascular Imaging Laboratory, Department of Radiology, University of Washington, 850 Republican Street, Box 358050, Seattle, WA 98109 USA; 2 BioMolecular Imaging Center, Department of Radiology, University of Washington, Seattle, WA USA; 3 Institute of Science and Technology for Brain-Inspired Intelligence, Fudan University, Shanghai, China; 4 Carle Illinois College of Medicine, University of Illinois at Urbana-Champaign, Champaign, IL USA; 5 Department of Electrical and Computer Engineering, University of Washington, Seattle, WA USA; 6 Department of Surgery, University of Washington, Seattle, WA USA

**Keywords:** Cerebrovascular disorders, Neurovascular disorders, Magnetic resonance imaging, Cognitive ageing, Neuro-vascular interactions

## Abstract

Developing novel risk markers for vascular contributions to cognitive impairment and dementia is important. This study aimed to extract total length, branch number and average tortuosity of intracranial distal arteries (A2, M2, P2 and more distal) from non-contrast enhanced magnetic resonance angiography (NCE-MRA) images, and explore their associations with global cognition. In 29 subjects (aged 40–90 years) with carotid atherosclerotic disease, the 3 intracranial vascular features on two NCE-MRA techniques (i.e. time of flight, TOF and simultaneous non-contrast angiography and intraplaque hemorrhage, SNAP) were extracted using a custom-developed software named iCafe. Arterial spin labeling (ASL) and phase contrast (PC) cerebral blood flow (CBF) were measured as references. Linear regression was performed to study their associations with global cognition, measured with the Montreal Cognitive Assessment (MoCA). Intracranial artery length and number of branches on NCE-MRA, ASL CBF and PC CBF were found to be positively associated with MoCA scores (*P* < 0.01). The associations remained significant for artery length and number of branches on NCE-MRA after adjusting for clinical covariates and white matter hyperintensity volume. Further adjustment of confounding factors of ASL CBF or PC CBF did not abolish the significant association for artery length and number of branches on TOF. Our findings suggest that intracranial vascular features, including artery length and number of branches, on NCE-MRA may be useful markers of cerebrovascular health and provide added information over conventional brain blood flow measurements in individuals with cognitive impairment.

## Introduction

Vascular dysfunction and damage are known to play important roles in the development of cognitive impairment and dementia^1,2^. However, the exact nature of the link between vascular health and cognitive performance still needs to be further clarified. In particular, given the wide spectrum of vascular diseases, it is unclear which components are more related to, or have earlier predictive value for, cognitive decline. At the moment, identifying novel risk markers has been considered to be a critical component in order to advance the field of vascular contributions to cognitive impairment and dementia^1^.

Hemodynamic abnormalities such as lower and dysregulated brain blood flow, and white matter hyperintensity (WMH) seen on magnetic resonance (MR) T2 weighted image that is believed to be a surrogate for small vessel disease^3^, have been shown to be associated with cognitive decline and dementia^4–6^. However, the underlying mechanism of such association needs further study. Although several mature imaging techniques are available for brain blood flow measurement in humans, such as single-photon emission computed tomography (SPECT), positron emission tomography (PET), arterial spin labeling (ASL) magnetic resonance imaging (MRI), transcranial doppler, and phase contrast (PC) MRI^7–9^, an effective and routinely applicable technique for characterizing the hemodynamic abnormalities in subjects with cognitive impairment is yet to be established.

Recently, features of cerebral vasculature, such as artery length and number of branches, obtained from brain non-contrast enhanced magnetic resonance angiography (NCE-MRA) imaging, such as time of flight (TOF)^10^ and simultaneous non-contrast angiography and intraplaque hemorrhage (SNAP)^11^, have been proposed as potentially useful indicators of brain blood flow^12,13^. This is mainly due to the fact that artery visibility on NCE-MRA, and thus the derived relevant intracranial vascular features, are affected by blood flow. Importantly, the blood flow information indirectly represented by the NCE-MRA intracranial vascular features, such as artery length and number of branches, are different from blood flow information of the proximal arteries (measured by e.g. PC MRI) or brain parenchyma (measured by e.g. ASL MRI), given that these features are mainly derived from the distal arteries (A2, M2, P2 and more distal). Moreover, such NCE-MRA intracranial vascular features may also be affected by other factors beyond blood flow, such as wall thickening-induced luminal narrowing in the distal arteries. Therefore, these NCE-MRA intracranial vascular features have the potential to provide unique information of blood flow and luminal narrowing in distal arteries. Furthermore, measurement of these NCE-MRA intracranial vascular features has been shown to be highly reproducible^14^. This may be another advantage of these NCE-MRA intracranial vascular features over other brain blood flow measurement techniques which may have relatively large variability due to physiological noise or insufficient spatial resolution^7,15,16^. Overall, due to their unique blood flow information and the reproducible measurements, NCE-MRA intracranial vascular features, such as artery length and number of branches, may potentially provide novel insight into the cerebrovascular condition of subjects with cognitive impairment.

Therefore, this study aims to explore the associations of intracranial vascular features, including artery length, number of branches and average tortuosity, measured from TOF-MRA or SNAP-MRA, with global cognitive function measured by the Montreal Cognitive Assessment (MoCA). Conventional blood flow measurements with ASL and 3D PC were also performed in order to test whether the measured intracranial vascular features’ associations with global cognitive function are independent of conventional blood flow measurements.

## Materials and methods

### Study population

The population of this study was 29 consecutive subjects already enrolled in an ongoing study of carotid atherosclerosis. The inclusion and exclusion criteria have been described previously^17^. In brief, all included subjects had carotid atherosclerosis with a stenosis > 15% and < 80% according to clinical ultrasound, CT or MRI, and were asymptomatic with regard to carotid disease.

All subjects underwent carotid MRI (for the ongoing carotid study) and conventional brain MRI (i.e. T1W, T2W, FLAIR and DWI) during the same scan session, followed by brain vascular MRI (i.e. TOF and SNAP) and blood flow MRI (i.e. ASL and PC) on a different day. Global cognitive function was assessed using the MoCA test prior to the brain vascular MRI and the two were performed on the same day. Clinical data for the subjects, including age, sex, body mass index, use of antihypertensive drugs, systolic blood pressure, diastolic blood pressure, history of diabetes mellitus, stroke or transient ischemic attacks, and smoking, were collected. The study was approved by University of Washington Institutional Review Board and was performed in accordance with the Helsinki Declaration. Informed consent was obtained from all participants prior to enrollment.

### MR imaging

Carotid stenosis was measured by a radiologist on 3D black blood carotid MR vessel wall images using the North American Symptomatic Carotid Endarterectomy Trial criteria (NASCET), as described in a previous study by Zhao et al.^18^.

MR imaging was performed on a Philips Ingenia 3.0 T CX MR system (Philips Healthcare, Best, The Netherlands) equipped with a 32-channel head coil for brain imaging. In the brain vascular and blood flow session, 3D TOF, 3D SNAP, 3D ASL and 3D PC were performed. A recently developed sequence named iSNAP^19^ was also performed to obtain whole-brain MPRAGE-like 3D T1W structural images for image registration. In the conventional brain MRI scan session, 2D T2W FLAIR images were acquired for detecting cerebral WMHs, which is a factor that may contribute to cognitive impairment and will be considered in the regression analyses mentioned later.

**Figure 1:**
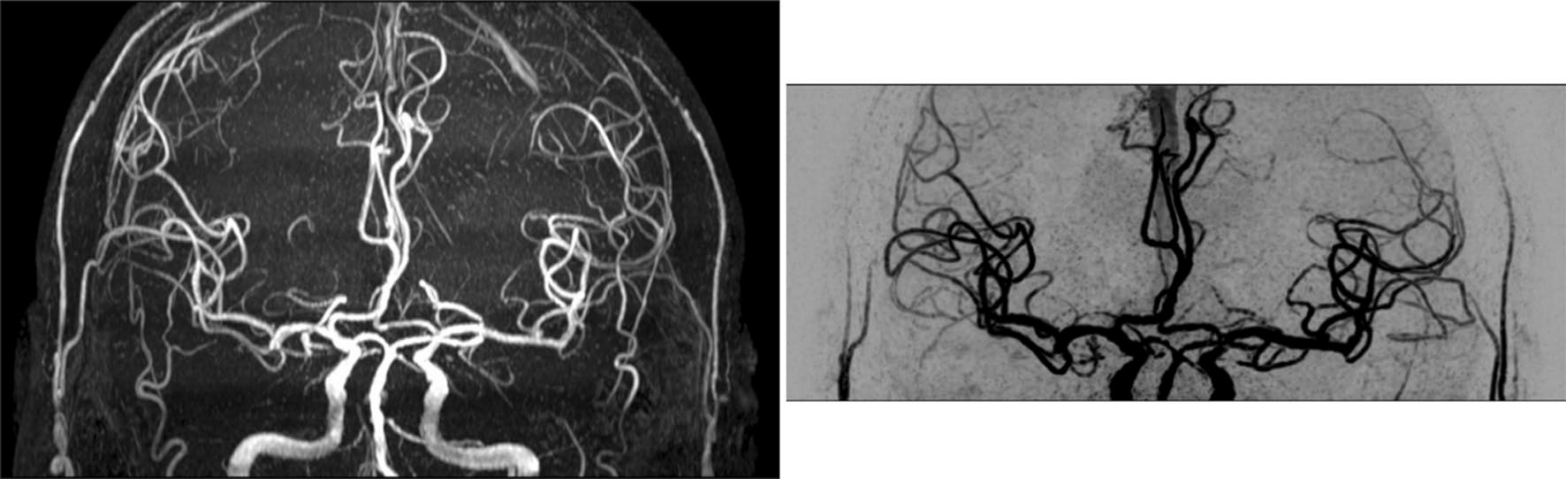
Coronal maximum/minimum intensity projection of TOF (left) and SNAP (right), showing spatial coverage in the feet-head direction.

The main imaging parameters of the MR sequences were as follows. TOF: FOV 190 × 180 × 105 mm^3^, 6 slabs, voxel size 0.5 × 0.5 × 1 mm^3^, total acquisition time 6 min 37 s; SNAP: FOV 180 × 180 × 70 mm^3^, voxel size 0.8 mm isotropic, total acquisition time 3 min 45 s; ASL: using pseudo-continuous labeling scheme, FOV 240 × 240 × 125 mm^3^, voxel size 3 × 3 × 5 mm^3^, labeling duration 1800 ms, post labeling delay 2000 ms, total acquisition time 4 min 55 secs; PC: FOV 180 × 180 × 70 mm^3^, voxel size 0.5 × 0.5 × 1 mm^3^, encoding velocity 100 cm/s in all three directions, total acquisition time 5 min 4 s; iSNAP: FOV 204.8 × 179.2 × 144 mm^3^, voxel size 0.8 mm isotropic; 2D T2W FLAIR: FOV 230 × 230 mm^2^, in-plane resolution 1.28 × 1.28 mm^2^, slice thickness 4 mm, gap between slices 1 mm, 25 slices, total acquisition time 2 min. Note that the FOVs of the SNAP and 3D PC scans were purposely positioned upwards to cover more distal arteries, with the distance between circle of Willis and inferior edge of FOV being around 13 mm (Fig. 1)^17^. Other details of the protocols of TOF, SNAP, ASL and PC were described elsewhere^17^.

### Image analyses

**Figure 2:**
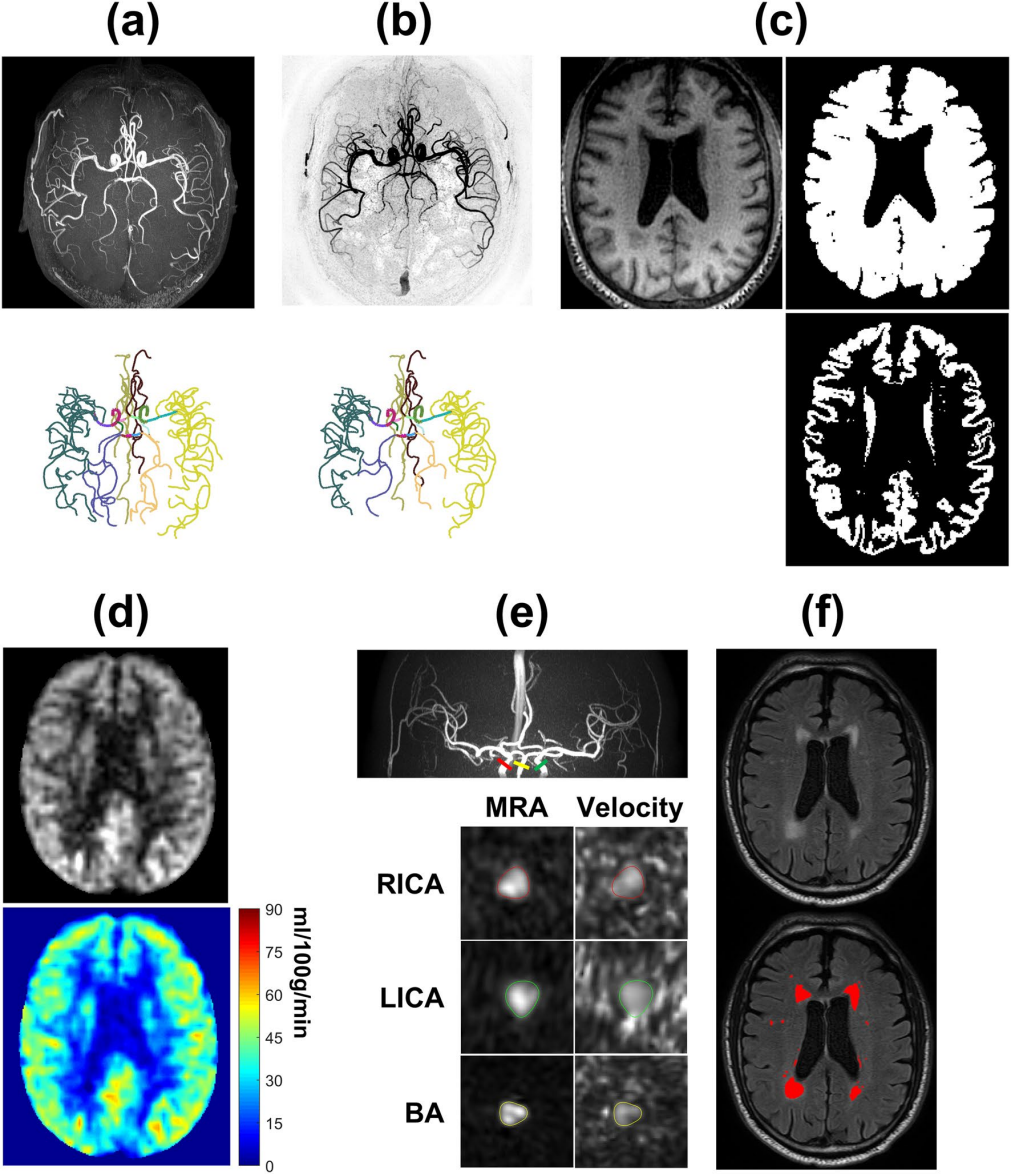
Illustration of image analyses. (**a**,**b**): TOF (**a**) and SNAP (**b**) MRA images (upper panels), and the corresponding traced and labeled intracranial arteries (bottom panels). (**c**): A representative slice of the T1-weighted image (upper left panel), binary brain mask (upper right panel) and gray matter mask (bottom right panel). (**d**): A representative slice of the ASL perfusion weighted image (upper panel) and the corresponding CBF map (bottom panel). (**e**): The MRA image (upper panel) obtained from PC complex difference image, and the cross-sectional PC complex difference images and PC velocity images of the RICA, LICA and basilar artery (bottom panel). The arterial contours were depicted on the PC complex difference image and then mapped to PC velocity image. (**f**): A representative slice of FLAIR image (upper panel) and the segmented white matter hyperintensity (red region).

Processing of TOF, SNAP, ASL and PC has also been detailed previously^17^. In brief, by using a custom-designed dedicated analysis tool named iCafe^20^, the intracranial arteries on TOF or SNAP images were semi-automatically traced to generate centerlines, which were subsequently manually labeled by a trained reviewer (Fig. 2a,b). Then the total length, number of branches, and average branch-wise tortuosity of the anterior cerebral arteries distal to A1, middle cerebral arteries distal to M1 and posterior cerebral arteries distal to P1 were calculated. To obtain the total arterial length, length of each arterial segment was first calculated by summing up the Euclidean distances between the centerline points and then added together over all segments. Number of branches is the number of arterial segments which start from a bifurcation and end in another bifurcation or termination in a vascular group. To achieve a fair comparison, the TOF images were cropped to obtain the same coverage as SNAP before calculating the vascular features^17^. The quantification of the above-mentioned intracranial vascular features using iCafe has been previously reported as highly reproducible^14^. iSNAP T1W images were used to perform brain segmentation (Fig. 2c) using SPM12 (https://www.fil.ion.ucl.ac.uk/spm). CBF maps (Fig. 2d) and mean gray matter CBF (in ml/100 g/min) were calculated from ASL images^8^. Cross sectional PC images were generated near the middle of internal carotid arteries and the basilar artery, and artery contours were drawn semi-automatically (Fig. 2e). Then, whole-brain mean PC CBF (in ml/100 g/min) was calculated as the total volume flow rate (i.e. mean velocity multiplied by area) of the two internal carotid arteries and the basilar artery, divided by the product of total brain volume (i.e. total volume of gray matter and white matter obtained from the brain segmentation) and brain density (assumed to be 1.06 g/ml)^16^.

WMHs were first automatically segmented based on FLAIR (Fig. 2f) and iSNAP T1W images, using a lesion segmentation tool (LST)^21^ combined with SPM12. Then, segmentation results were manually corrected by a radiologist using FSLeyes software (https://fsl.fmrib.ox.ac.uk/fsl/fslwiki/FSLeyes). The total whole brain WMH volume was then calculated.

### Statistics

Categorical variables are presented as frequencies and percentages, while continuous variables are presented as mean ± SD. To mitigate the influence of brain size, the TOF and SNAP intracranial artery length were normalized with the cube root of total brain volume, while the WMH volume was normalized with total brain volume. Due to overt skewness, the WMH volume was further log-transformed.

The associations between the different NCE-MRA intracranial vascular features (i.e. artery length, number of branches and tortuosity), blood flow measurements, WMH volume and MoCA scores were first analyzed with univariable linear regression, with the MoCA score used as a dependent variable and the NCE-MRA vascular feature, blood flow measurement or WMH volume, as an independent variable. Then, multivariable linear regressions that include potential confounders were performed. Considering that subjects included all had carotid atherosclerosis, the maximum stenosis of bilateral carotid arteries was included as a confounder. To identify other confounders, univariable linear regression between each clinical variable and MoCA score was performed, and only the clinical variables with a *P* < 0.1 were chosen as confounders. Additional multivariable linear regressions that adjust for WMH volume and the clinical variables were performed for the associations between the NCE-MRA vascular features or blood flow measurements and MoCA score.

Multivariable linear regressions that adjust for ASL/PC CBF and the clinical variables were also performed, in order to investigate whether the associations between the measured TOF or SNAP intracranial vascular features and MoCA scores are independent of conventional brain blood flow measurements.

In the above linear regression analyses, all continuous variables were standardized. The statistics were performed in Matlab 2019b (Mathworks, Natick, MA). A *P* value < 0.05 was considered statistically significant. Because of the exploratory nature of the analyses, no adjustment of P values due to multiple comparisons was performed.

## Results

MR imaging scans and MoCA tests were all successfully performed on the 29 enrolled subjects (aged 40–90 years). Clinical characteristics are shown in Table 1. The median time interval between the brain MRI and brain vascular MRI was 144 days (range: 63–323 days).

**Table 1:** Clinical characteristics of the enrolled subjects (N = 29).

Variable	Mean ± SD or N (%)
Age (year)	71.8 ± 9.9
Male	18 (62.1)
BMI (kg/m^2^)	27.4 ± 4.1
History of stroke or TIA	15 (51.7)
Use of antihypertensive drug	20 (69.0)
Systolic blood pressure (mm Hg)	142.9 ± 11.6
Diastolic blood pressure (mm Hg)	77.8 ± 9.9
Diabetes mellitus	3 (10.3)
Smoking	11 (37.9)
Stenosis of right carotid (%)	28.86 ± 16.97
Stenosis of left carotid (%)	33.60 ± 20.18
Maximum carotid stenosis (%)	42.54 ± 17.00

BMI was not available for one subject, and systolic and diastolic blood pressure were not available for one subject. BMI: body mass index; TIA: transient ischemic attack.

Results of the univariable and multivariable linear regression analyses for associations between the different NCE-MRA vascular features, blood flow measurements, WMH volumes and MoCA scores are shown in Table 2. Among the clinical characteristics, age (*P* = 0.009), use of antihypertensive drugs (*P* = 0.049) and systolic blood pressure (*P* = 0.047) were found to have a *P* < 0.1 in the univariable linear regression analysis for association with MoCA scores, and therefore were included as confounding factors in the multivariable linear regression model.

**Table 2:** Associations of different intracranial vascular features on NCE-MRA, brain blood flow measurements, WMH volume with MoCA score (N = 29).

	Univariable linear regression			Multivariable linear regression					
				Model 1			Model 2		
	*β*	*P*	Adjusted *R*^2^	*β*	*P*	Adjusted *R*^2^	*β*	*P*	Adjusted *R*^2^
TOF, artery length	0.605	** < 0.001**	0.343	0.513	**0.004**	0.474	0.516	**0.004**	0.453
SNAP, artery length	0.520	**0.004**	0.244	0.432	**0.044**	0.358	0.481	**0.039**	0.341
TOF, number of branches	0.637	** < 0.001**	0.383	0.559	**0.002**	0.512	0.559	**0.002**	0.489
SNAP, number of branches	0.581	** < 0.001**	0.313	0.543	**0.010**	0.431	0.647	**0.006**	0.440
TOF, average tortuosity	−0.243	0.203	0.024	−0.167	0.343	0.256	−0.175	0.353	0.222
SNAP, average tortuosity	0.108	0.578	−0.025	−0.015	0.933	0.225	−0.011	0.952	0.189
ASL CBF	0.526	**0.003**	0.250	0.321	0.107	0.313	0.350	0.105	0.286
PC CBF	0.480	**0.008**	0.202	0.352	0.051	0.351	0.425	**0.037**	0.343
WMH volume	−0.178	0.355	−0.004	−0.025	0.896	0.225			

Significant values are in bold.

Systolic blood pressure was not available for one subject. Therefore, the same size for Model 1 and Model 2 was 28. Model 1 was adjusted for maximum carotid stenosis, age, use of antihypertensive drug and systolic blood pressure; Model 2 was Model 1 plus adjustment for WMH volume.

ASL CBF, PC CBF, and all of the measured NCE-MRA vascular features except average tortuosity, had positive associations with MoCA scores in the univariable linear regression analysis; and the associations for the NCE-MRA vascular features remained statistically significant after adjusting for clinical confounding factors (Model 1 in Table 2); except for ASL CBF, the associations remained statistically significant after adjusting for clinical confounding factors and WMH volumes (Model 2 in Table 2). The strength of association (i.e. the adjusted R-squared) was highest for artery length and number of branches on TOF. No association between WMH volumes and MoCA scores was observed in this study.

The associations of artery length and number of branches on TOF with MoCA scores remained statistically significant after adjusting for ASL CBF or PC CBF (Table 3). Regarding the SNAP sequence, associations between artery length and MoCA scores disappeared after adjusting for ASL CBF or PC CBF, while number of branches still had a marginally significant association with MoCA scores (Table 3).

**Table 3:** Associations of intracranial vascular features on TOF/SNAP with MoCA cognitive score after adjusting for ASL/PC CBF (N = 28).

	Adjusted for ASL CBF			Adjusted for PC CBF		
	*β*	*P*	Adjusted *R*^2^	*β*	*P*	Adjusted *R*^2^
TOF, artery length	0.484	**0.018**	0.452	0.441	**0.014**	0.492
SNAP, artery length	0.366	0.220	0.331	0.329	0.129	0.392
TOF, number of branches	0.527	**0.007**	0.493	0.496	**0.012**	0.502
SNAP, number of branches	0.600	**0.047**	0.406	0.441	0.056	0.431
TOF, average tortuosity	−0.182	0.283	0.319	−0.164	0.318	0.352
SNAP, average tortuosity	−0.046	0.792	0.282	−0.037	0.830	0.321

Significant values are in bold.

In addition to ASL CBF or PC CBF, we also adjusted for maximum carotid stenosis, age, use of antihypertensive drug and systolic blood pressure.

**Figure 3:**
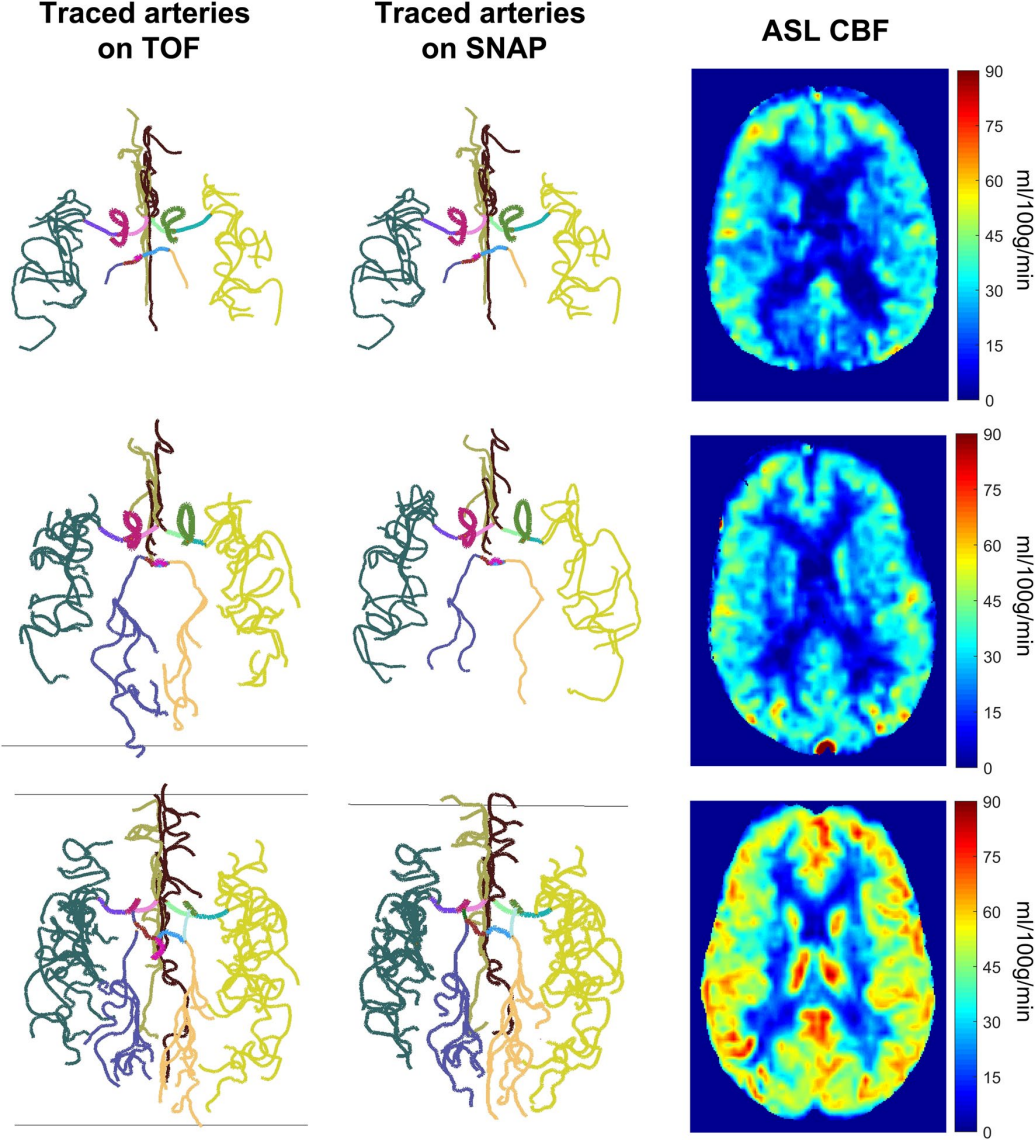
Traced intracranial arteries on TOF and SNAP, and ASL CBF maps of 3 representative subjects with different MoCA scores. The first subject (the first row) was a 77-year-old male with a MoCA score of 18 and a PC CBF of 34.0 ml/100 g/min; the second subject (the second row) was a 77-year-old male with a MoCA score of 25 and a PC CBF of 34.1 ml/100 g/min; the third subject (the third row) was a 40-year-old male with a MoCA score of 30 and a PC CBF of 44.5 ml/100 g/min.

Vessel tracing results on TOF and SNAP, ASL CBF map and PC flow measurements of three representative subjects, who had low, moderate, and high MoCA scores, respectively, are shown in Fig. 3.

## Discussion

This work explored the potential of the intracranial vascular features such as artery length and number of branches on NCE-MRA as imaging biomarkers of cognitive impairment. Similar to ASL CBF and PC CBF, we found that intracranial artery length and number of branches on either TOF or SNAP images were significantly associated with MoCA scores, before and after adjusting for clinical covariates. Additionally, the strength of associations was higher for artery length and number of branches on TOF than for other vascular features and brain blood flow measurements. Furthermore, NCE-MRA vascular features, especially artery length and number of branches on TOF, were found to have associations with MoCA scores independent of ASL CBF and PC CBF. These results suggest that the measured intracranial vascular features on NCE-MRA may be useful imaging biomarkers for studying cognitive impairment, and that intracranial artery length and number of branches on TOF have potential to provide added information regarding cerebrovascular health over conventional blood flow measurements by ASL and PC.

In this study, intracranial artery length and number of branches on NCE-MRA are not a measure of real total artery length or number of branches in the brain. Instead, they are surrogates of factors that would affect luminal signal and visibility of distal arteries (A2, M2, P2 and more distal) on NCE-MRA. According to previous theoretical simulation studies^22,23^ and an in vivo validation study^13^, blood flow velocity plays a critical role in affecting arterial signal intensity and visibility of distal arteries on both TOF and SNAP sequences. A smaller velocity and a longer traveling length of blood flow spins will lead to a larger number of RF pulse experienced by the spins, and thus more magnetization saturation and decreased artery visibility. In-plane flow is a typical factor that can increase traveling length and reduce blood signal intensity, and therefore may contribute to a decrease in calculated artery length and number of branches. This indicates that the effect of in-plane flow has been implicitly considered in the observed associations between artery length/number of branches and cognitive impairment. Therefore, artery visibility on TOF and SNAP is dependent on overall blood flow velocity in the whole traveling path. Due to the use of multi-slab acquisition in TOF (17.5 mm-thick per slab in this study), artery visibility on TOF is more associated with local blood flow velocity than the artery visibility on SNAP^22^. Apart from blood flow, other conditions, especially luminal diameter, may also affect the visibility of the distal brain arteries on NCE-MRA. Such vessel conditions of the distal brain arteries have rarely been studied, which may be due to the spatial resolution limit of MRI. Therefore, intracranial artery length and number of branches on NCE-MRA are likely to be a measure that combines multiple aspects of cerebrovascular health, with the contribution of each aspect differing between NCE-MRA techniques.

In this study, both conventional brain blood flow imaging (i.e. ASL and PC) and artery length and number of branches on NCE-MRA were observed to be associated with global cognition (Table 2). The association on PC CBF and ASL CBF are in line with findings from previous cognition-related studies that also used PC or ASL MRI to measure brain blood flow^4,24,25^. Given the established correlation between intracranial vasculature features on TOF or SNAP and ASL or PC^12,13^, the association between TOF or SNAP intracranial artery length/number of branches and MoCA scores can be largely explained by the inherent sensitivity of TOF and SNAP sequences to blood flow. Importantly, although the TOF and SNAP sequences were not originally designed to measure brain blood flow, their associations with MoCA are not smaller than ASL CBF and PC CBF (Table 2). This suggests that intracranial vascular features such as artery length and number of branches on NCE-MRA may be useful in assessment of cognitive impairment. This application can be considered an extension of the utility of brain NCE-MRA, which is conventionally used to assess morphology of large intracranial vessels. Such an extension of utility may be of great clinical value, given that NCE-MRA, especially TOF-MRA, is widely used and available in clinical practice.

A novelty of this study is that we performed two different NCE-MRA techniques and two different conventional brain blood flow measurements on the same subjects, thus enabling comparison of the strength of association with MoCA scores, which has not been performed in previous studies. The stronger and independent association of intracranial artery length and number of branches on TOF with MoCA scores, as compared to intracranial artery length and number of branches on SNAP, ASL CBF and PC CBF, may be explained by the difference in imaging mechanisms. As discussed above, artery length and number of branches derived from TOF are mainly affected by local flow velocity of the distal arteries. On the contrary, visibility of the distal arteries on SNAP is affected by blood spins’ traveling time starting from very proximal extracranial internal carotid and vertebral arteries^23^. ASL CBF is a measure of blood volume passing through a given amount of tissue per unit of time, and is implemented with an image subtraction procedure^8^. PC MRI measures blood flow velocity at large vessels by using pairs of bipolar gradients^9^, and whole-brain mean CBF is obtained by integrating velocity over the cross section of all feeding arteries, which is then divided by brain volume. Note that the iSNAP sequence, which was used for brain segmentation in this study, is an emerging NCE-MRA technique that allows simultaneous generation of whole-brain T1W structural MRI and multiple vascular image contrasts^19^, and may be a promising tool for studying the link between vascular diseases and cognitive impairment. However, because of imaging parameter inconsistency over subjects and the lack of a mature dedicated post-processing method, iSNAP was not used to analyze the intracranial vascular features in this study.

Our findings suggest the importance of local blood flow velocity in distal brain arteries, as reflected by TOF intracranial artery length and number of branches, in assessing cognitive impairment. Another possible explanation for the independent association of the two TOF intracranial vascular features with MoCA scores is that TOF intracranial artery length and number of branches reflects cerebrovascular conditions beyond blood flow, such as luminal narrowing of distal arteries, while SNAP intracranial artery length and number of branches, as well as ASL CBF and PC CBF are strong indicators of brain blood flow. This explanation seems to be supported by the previous observation that TOF intracranial artery length has a weaker correlation with ASL CBF and PC blood flow than SNAP intracranial artery length^13^. In the future, dedicated studies are needed to explore the independent contribution of blood flow and other vascular conditions to vessel visibility in TOF images, for example by using ultra high-field MRI to achieve higher spatial resolutions for imaging diameter and flow in distal small arteries.

This study has several limitations. First, the sample size is relatively small, which may lead to insufficient power for observing some potential links between the studied imaging markers and MoCA scores. Second, the facts that the included subjects all had carotid atherosclerosis, the mean age was relatively high, and the possibility of a wide variance in clinical MRA protocols, may limit the generalizability of our findings. Third, while we found no correlation between WMH and MoCA scores, we cannot generalize this finding due to the time interval between FLAIR imaging, which was used to characterize white matter lesions, and the blood flow imaging and MoCA testing in this study. This may introduce bias in the multivariable linear regression analyses. Fourth, due to the cross-sectional nature of the study, we did not explore the predictive value of the imaging markers for cognitive impairment. Fifth, this study mainly focused on features of distal intracranial arteries (A2, M2, P2 and more distal) on NCE-MRA, while other vascular conditions, such as intracranial arterial stenosis, vulnerable atherosclerotic plaque, calcification and vascular compliance, may also be associated with cognitive impairment. Future studies are needed to explore the interplay of these different vascular measurements and their individual contribution to cognitive impairment. Sixth, the P values were not corrected for multiple comparisons. Further validation of the findings with a larger sample size and prospective studies in the future is needed.

## Conclusion

Intracranial artery length and number of branches on TOF and SNAP MRA are associated with MoCA scores, suggesting that these intracranial vascular features on NCE-MRA may be useful markers of cerebrovascular health in cognitive impairment. Furthermore, the associations of intracranial artery length and number for branches on TOF with MoCA scores were independent of carotid stenosis, age, systolic blood pressure, use of anti-hypertensive drugs, ASL CBF and PC CBF, suggesting that they may provide added information about cerebrovascular health over conventional blood flow measurements.

## Data Availability

The datasets used and/or analyzed during the current study are available from the corresponding author on reasonable request. Software and versions used in the study: iCafe 1.4.4: iCafe (IntraCranial Artery Feature Extraction) is a research tool for academic use only. Readers can obtain it by contacting Mr. Zach Miller (zach1@uw.edu), manager of vascular imaging laboratory (VIL) at University of Washington, Seattle. SPM 12: available from https://www.fil.ion.ucl.ac.uk/spm/. FSLeyes 0.30.0: available from https://fsl.fmrib.ox.ac.uk/fsl/fslwiki/FSLeyes.
